# Sex differences in the traumatic stress response: PTSD symptoms in women recapitulated in female rats

**DOI:** 10.1186/s13293-018-0191-9

**Published:** 2018-07-05

**Authors:** Apryl E. Pooley, Rebecca C. Benjamin, Susheela Sreedhar, Andrew L. Eagle, Alfred J. Robison, Michelle S. Mazei-Robison, S. Marc Breedlove, Cynthia L. Jordan

**Affiliations:** 1 Neuroscience Program, Michigan State University, 108 Giltner Hall, 293 Farm Lane, East Lansing, MI 48824 USA; 2 Department of Physiology, Michigan State University, 2201 BPS, 567 Wilson Rd, East Lansing, MI 48824 USA

**Keywords:** Sex differences, Single prolonged stress, Predator exposure, HPA axis, Post-traumatic stress disorder

## Abstract

**Background:**

Post-traumatic stress disorder (PTSD) affects men and women differently. Not only are women twice as likely as men to develop PTSD, they experience different symptoms and comorbidities associated with PTSD. Yet the dearth of preclinical research on females leaves a notable gap in understanding the underlying neuropathology of this sex difference.

**Methods:**

Using two standard measures of PTSD-like responses in rats, the acoustic startle response (ASR) and dexamethasone suppression test (DST), we tested the effects of traumatic stress in adult male and female rats using two rodent models of PTSD, single prolonged stress and predator exposure. We then examined the neural correlates underlying these responses with cFos and glucocorticoid receptor immunohistochemistry in brain regions implicated in the traumatic stress response.

**Results:**

We now report that adult male and female rats across two models of PTSD show consistent sex-specific responses that recapitulate fundamental differences of PTSD in men and women. Trauma-exposed males showed the well-established hyper-responsive phenotype of enhanced ASR and exaggerated negative feedback control of the hypothalamic-pituitary-adrenal axis, while the same traumatic event had little effect on these same measures in females. Dramatic sex differences in how trauma affected cFos and glucocorticoid receptor expression in the brain lend further support to the idea that the trauma response of male and female rats is fundamentally different.

**Conclusions:**

Two standard measures, ASR and DST, might suggest that females are resilient to the effects of traumatic stress, but other measures make it clear that females are *not* resilient, but simply respond differently to trauma. The next important question to answer is why. We conclude that males and females show fundamentally different responses to trauma that do not simply reflect differences in resilience. The divergent effects of trauma in the brains of males and females begin to shed light on the neurobiological underpinnings of these sex differences, paving the way for improved diagnostics and therapeutics that effectively treat *both* men and women.

**Electronic supplementary material:**

The online version of this article (10.1186/s13293-018-0191-9) contains supplementary material, which is available to authorized users.

## Background

The neurobiology of sex differences in post-traumatic stress disorder (PTSD) is not understood [1]. The prevalence of PTSD is twice as high in women compared to men, despite an overall lower risk for women to experience a traumatic event [2, 3]. While men and women experience different types of trauma at different rates, and women have an increased risk for PTSD following assaultive trauma, trauma type alone does not account for this sex difference in PTSD prevalence [2, 4, 5]. This sex difference in PTSD prevalence is evident even when men and women experience the same type of trauma, such as accidents, terrorism, and natural disaster [1]. Furthermore, regardless of trauma type or prevalence, women experience more chronic PTSD and different symptoms and comorbidities than men [3, 6–9]. This evidence suggests sex differences in the underlying neurobiology of the traumatic stress response, yet our current understanding of PTSD is based on males. Recent reviews emphasize the need to examine the neurobiology behind sex differences in PTSD [10], but this call has largely not been met. While the animal literature is replete with reports showing that stress affects males and females differently, and often with opposing effects on behavior, physiology, and the brain [11, 12], focus has been on acute or chronic stress as models of anxiety and depression, with little attention given to whether males and females respond differently to traumatic stress.

Two well-validated and commonly used rodent models of PTSD are single prolonged stress (SPS) and predator exposure (PredX). To date, over 200 SPS studies have been published [13], but only one directly compared males and females [14]. Similarly, only a handful of the > 100 published PredX studies have examined sex differences [15–17]. The SPS model uses physical and chemical stressors (restraint, forced swim, and ether) whereas the PredX model uses exposure to a natural predator (live cat). Exposure (but without direct contact) to a live cat elicits higher proportions of affected rats than predator odor exposure [18], and thus, most closely matches the intensity of the SPS stress paradigm, leading to comparable proportions affected. We chose these two models not only because of the similarities in the apparent impact of the trauma, but also because the stressors used in each model are sufficiently different to allow us to make inferences about the likely generalizability of any sex differences we discover. Fully understanding PTSD requires researchers to directly compare males and females exposed to the same types of stressors under the same conditions, and doing so with two different types of stressors can address whether sex differences in the traumatic stress response are stressor specific or are likely core attributes of traumatic stress in men versus women. Such controlled studies will enable investigators to identify the critical factors contributing to, and thus, the neurobiological underpinnings of, sex-specific responses to traumatic stress. The studies described in this report begin to address this issue.

Presumed core attributes of PTSD include hyper-responsiveness to stressful stimuli, indicated by an enhanced acoustic startle response (ASR) and an exaggerated negative feedback response of the hypothalamic-pituitary-adrenal (HPA) axis revealed by the dexamethasone suppression test under acute stress, leading to chronic hypocortisolism [19]. Such responses to trauma are readily observed in men with PTSD [20] and trauma-exposed male rodents [21]. However, whether these same readouts reflect the effects of trauma in females is much less clear [22]. Indeed, more than half of women with PTSD do *not* show the male-typical increase in negative feedback control of the HPA axis [23, 24]. Similarly, women with PTSD are less likely to show enhanced ASR, and in some cases, show *diminished* startle [25]. Current diagnostic criteria and clinical practices have not been revised to reflect these sex differences in the trauma response, largely due to a lack of basic research on females.

We embarked on a series of studies to examine the response of male and female rats exposed to the same traumatic stress, either SPS or PredX. We began by directly comparing intact males to intact, normally cycling females, since sex differences in fear conditioning are present regardless of estrogen levels in women with PTSD [26]. Moreover, sex differences in rat HPA axis activity [27–30] and startle response [31] are not driven by changes in the female hormone cycle. We find robust sex differences in the traumatic stress response at every level of analysis, from behavior to the stress hormone response to cellular measures in the brain. Taken together, these findings begin to uncover novel neurobiological mechanisms underlying sex-specific responses to trauma.

## Methods

### Animals

Eight-week-old adult Sprague-Dawley male and female rats (total *n* = 112) were purchased from Charles River (Wilmington, MA, USA) and housed with 12-h reversed light-dark cycle, ad lib food and water. Cage bedding was changed weekly, and no testing was conducted on days of cage changes. Rats were housed in same-sex pairs on the day of arrival and handled 3 min daily for 1 week before any testing or stress exposure. All behavior tests were conducted in the dark phase ≥ 2 h of dark. Female rats were freely cycling and assigned to treatment groups without regard to estrous cycle stage. All animal procedures and care met or exceeded the NIH guidelines and were approved by Michigan State University Institutional Animal Care and Use Committee.

### Study design

**Fig. 1:**
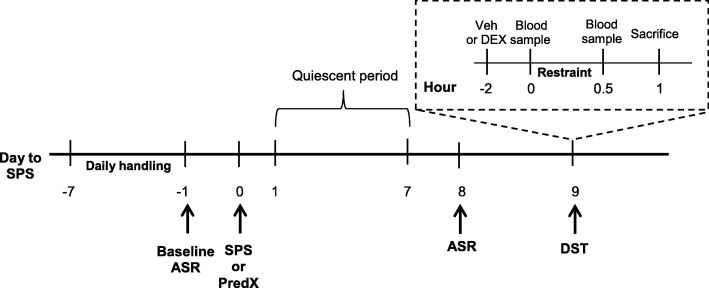
Timeline of experimental procedures. Experimental timeline begins with daily handling 1 week before (− 7) single prolonged stress (SPS) or predator exposure (PredX) and baseline acoustic startle response (ASR) testing the day before (− 1) SPS or PredX. Rats are left undisturbed for 1 week after SPS or PredX, with post-stress ASR assessed 8 days later, and dexamethasone suppression test (DST) 9 days later

Male and female rats were exposed to a traumatic stress paradigm or a no-stress control condition, in a 2 × 2 design with sex and stress being the two main factors. The experimental timeline (Fig. 1) began with daily handling of rats for 1 week before SPS or PredX. Baseline ASR testing was conducted the day before SPS or PredX, and rats were left undisturbed for 1 week after stress. Post-stress ASR was assessed 8 days later, and dexamethasone (DEX) suppression test (DST) 9 days later. Each experiment was conducted using multiple independent cohorts of rats, with equal numbers of rats representing all treatment groups (minimum of two rats per group) in a single cohort yoked through the entire experiment. The number of cohorts required was determined a priori by a power analysis using effect sizes obtained from preliminary studies to determine the number of rats required per group to achieve 0.80 statistical power, and data from each cohort in a single experiment were collapsed after confirming via ANOVA no significant cohort effects.

### Acoustic startle response

Rats were placed in a Plexiglas tube attached to an accelerometer inside a dark, soundproof chamber (SR-Lab, San Diego Instruments, CA, USA) and allowed to acclimate for 5 min (68-dB background noise) before delivery of a startle stimulus (50-ms burst of 110-dB white noise every 30 s for 15 min), as previously described [21]. The chamber and Plexiglas tube were cleaned with 70% ethanol between each test. Peak whole-body startle response was recorded every 1 ms for 100 ms, beginning with each startle stimulus. The average peak value for each rat was normalized to body weight. Baseline measures were taken on the day before stress exposure, with rats randomly selected and counterbalanced by group and cagemates tested simultaneously in two separate chambers. Rats were then assigned to control or stress groups so that each group had equal average ASR [32]. ASR testing was repeated 8 days after stress exposure with individual rats tested in the same order as baseline ASR. This repeated-measures design is important because inter-individual differences in ASR can be large, but startle response is a stable trait within an individual, both in humans and rats, so any effects of trauma can be detected by comparing changes between pre- and post-test [33, 34].

### SPS paradigm

All rats were singly housed immediately before exposure to SPS or control conditions to eliminate any sex-specific effects of social support on the stress response. We were particularly concerned that pair housing might mask an effect of trauma in females [35]. SPS consists of a psychological stressor (2-h tube-restraint stress, Braintree Scientific, Braintree, MA, USA), a physiological stressor (20-min group forced swim in 24 °C water, *n* = 6 same-sex rats per 75-l tub with 28 cm water depth), and a chemical stressor (brief exposure to diethyl ether until immobile and lacking toe-pinch response), as previously described [36]. Rats were given a 15-min rest period in their home cages after the forced swim before ether exposure. Ether is used as a chemical/endocrinological stressor to recapitulate an important phenotypic aspect of PTSD and is not used as an anesthetic. Control rats were also similarly removed from the vivarium for 2.75 h. Rats were left undisturbed for 1 week after SPS, a requisite delay for the long-lasting PTSD responses to develop [21].

### PredX paradigm

Rats were placed in individual wedge-shaped enclosures of a circular Plexiglas “pie restrainer” (Braintree Scientific, Braintree, MA, USA) on which cat food was smeared and placed inside a Plexiglas arena (60 cm^3^) containing a live female cat for 1 h, as previously described [16]. This paradigm allows rats to be exposed to the cat without physical contact. All rats were singly housed immediately before the PredX and control procedure. Control rats were removed from the vivarium for 1 h but not put in the restrainer nor exposed to the cat and were housed in a separate room to prevent exposure to possible residual predator scent on PredX rats. Rats were left undisturbed for 1 week after PredX to allow the acute stress responses to resolve and the long-term responses to trauma develop.

### Dexamethasone suppression test (DST)

The DST is a tool used in clinical and experimental studies to detect disruption of the HPA axis [37]. Dexamethasone (DEX) is a pituitary GR agonist [38] that, when administered hours before a stressful stimulus, diminishes any subsequent cortisol/corticosterone (CORT) response via activation of negative feedback control of the HPA axis. Men with PTSD typically show exaggerated suppression of CORT with DEX compared to healthy controls [39]. To assess the strength of negative feedback control of the HPA axis in response to acute restraint stress, the DST was performed 9 days post-SPS/PredX, as previously described [21]. Rats from each group were randomly assigned to receive either DEX or vehicle. Dexamethasone (Sigma-Aldrich, St. Louis, MO, USA) was dissolved with ethanol and diluted to 5% in sterile saline. Low-dose DEX (0.05 mg/kg, i.p.) or vehicle was administered 2 h prior to 30 min tube-restraint. In rats, this dose of DEX has been determined to produce a submaximal suppression of CORT responses to acute restraint stress that allows for detection of exaggerated DEX suppression of CORT [40]. Tail-nick blood samples were collected at 0 and 30 min of restraint. Blood samples were collected in the rats’ dark phase, matching the time of day across experimental groups. Plasma CORT levels were determined using an enzyme immunoassay kit. Rats were overdosed with pentobarbital (i.p.) after 30 min of restraint, then intracardially perfused with saline and 4% buffered paraformaldehyde, with brains harvested for staining.

### cFos and glucocorticoid receptor (GR) immunohistochemistry (IHC)

Brains were sectioned and labeled for cFos or GR using a peroxidase ABC kit (Vectastain Elite ABC Kit, Vector Labs, cat# PK-6200), and only rats that received vehicle injections (and not DEX) were used to map specific neuronal populations activated by acute restraint stress and those expressing GR. A rabbit IgG polyclonal cFos antiserum (1:10,000; Santa Cruz Biotech, cat# sc-52, Dallas, TX, USA) and diamenobenzidine was used to visualize cFos expression, as previously described [41]. GR IHC used the same basic protocol on alternate sections from the same brains, with a GR primary antiserum (1:2500; rabbit polyclonal IgG; Santa Cruz Biotech, cat# M-20, Dallas, TX, USA). Specificity of GR staining was confirmed by observing a loss of nuclear staining when the GR antiserum was preadsorbed with the immunizing peptide and observing the expected regional staining (e.g., in the dentate gyrus and CA1 but not CA3). Microscope analysis was conducted on a Zeiss Axioplan light microscope equipped with a video camera and MBF Stereo Investigator software (MBFBioscience, Williston, VT, USA). The number of cFos+ or GR+ neurons within specific brain regions was counted blind from four comparable sections per rat using unbiased stereological methods, and the number of immunopositive cells per cubic millimeter was quantified for each region by dividing the total number of cells counted within a region by the measured volume of that region. Anatomical position was determined using a stereotaxic atlas.

### Statistical analysis

All data were collected by experimenters blind to treatment group. All rats from all cohorts in a single experiment were included in the final statistical analysis, excepting the following exclusion criteria determined a priori: (1) if baseline CORT was not reduced in a DEX-treated rat *and* experimenter notes confirm a partial/problematic injection, that individual was excluded from *only* the DST analysis, and (2) if CORT levels did not increase (either 0 or negative change) after 30 min of restraint, that individual was excluded *only* from the DST analysis. These exclusion criteria resulted in the exclusion of 0–2 individuals from each treatment group for the DST. After confirming equal variance between treatment groups, two- or three-way ANOVAs were run for comparisons in rats. See Additional files 1 and 2 for all statistical tests performed. For brain measures, if no main effect or interactions of hemisphere were present, data were collapsed across hemisphere. The conservative Bonferroni test was used to correct for multiple tests to hold the probability of a type I error at 0.05.

## Results

### Male and female rats respond differently to single prolonged stress (SPS)

**Fig. 2:**
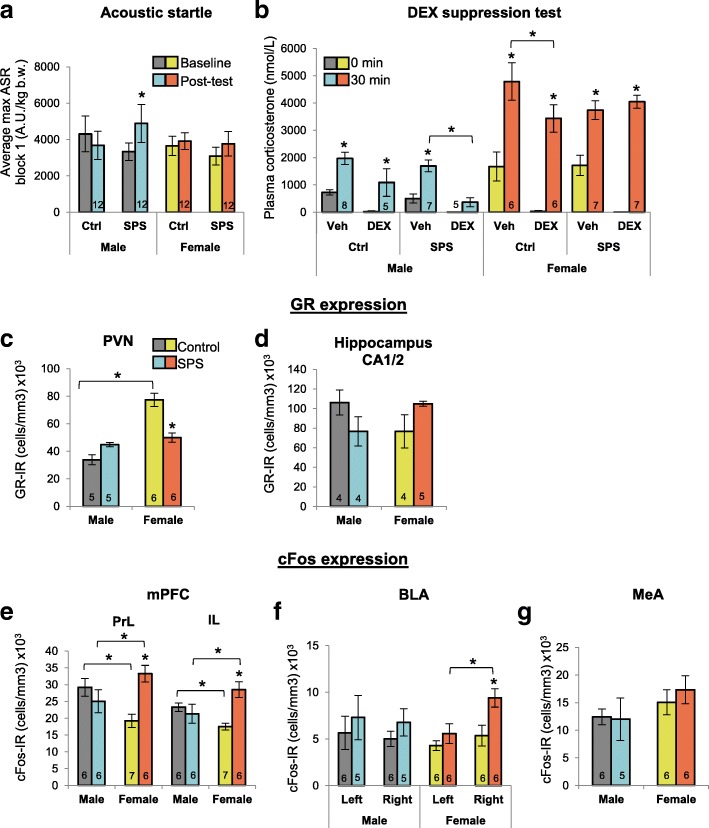
Single prolonged stress (SPS) affects males and females differently. **a** Exposure to SPS increased the acoustic startle response (ASR) in male but not in female rats. **b** Likewise, the dexamethasone (DEX) suppression test (DST) revealed an enhanced sensitivity to DEX only in SPS-exposed males, with DEX significantly lowering CORT levels after acute restraint stress compared to vehicle for SPS-exposed males. DEX treatment of control males failed to significantly reduce the CORT response. Surprisingly, CORT levels of SPS females after restraint stress were comparable, regardless of DEX treatment, suggesting DEX-nonsuppression, a characteristic of depression. Note that DEX lowered baseline CORT levels (0 min) in all groups, demonstrating its effectiveness in both sexes. CORT levels were significantly higher in females compared to males at both time points, as expected. **c**, **d** SPS had divergent effects on glucocorticoid receptor (GR) expression in the brain, with GR expression in the paraventricular nucleus of the hypothalamus (PVN) somewhat increased by SPS in males (*P* = .060) but significantly *decreased* in females. SPS also affected GR expression in hippocampal CA1/2, decreasing GR in males but increasing GR in females (sex*SPS interaction *P* = .050). GR expression in the PVN of control females was also higher compared to that of control males. **e**–**g** Surprisingly, SPS had no effect on the cFos response of males to restraint stress in the prelimbic (PrL) or infralimbic (IL) subregions of the medial prefrontal cortex (mPFC), basal lateral amygdala (BLA), nor medial amygdala (MeA), but significantly increased cFos responding of females to restraint stress in the PrL, IL, and right BLA of females. The cFos response also showed a sex difference in the mPFC, being lower in control females than control males. Data presented as mean ± SEM. Significance set at *P* < .05 (indicated by asterisk) for planned pairwise comparisons (Bonferroni). Refer to Additional file 1 for full statistical results

As expected, males exposed to SPS showed an enhanced acoustic startle response (ASR) (Fig. 2a) compared to their pre-stress baseline ASR (*P* = 0.017), replicating a well-established effect of SPS [21]. On the other hand, SPS had no effect on ASR in females (Fig. 2a). The dexamethasone (DEX) suppression test (DST) also revealed the expected enhanced sensitivity to DEX in SPS-exposed males, blocking the restraint-induced increase in circulating corticosterone (CORT) levels typical of control males (Fig. 2b). In contrast, females exposed to SPS showed a *reduced* sensitivity—DEX pretreatment, which competes with endogenous CORT for glucocorticoid receptor (GR) binding, failed to suppress the stress-related increase in CORT levels in SPS-exposed females (Fig. 2b). Notice however that for control females, this same dose of DEX effectively suppressed stress-induced increases in CORT levels and that DEX lowered baseline CORT levels (0 min) in all groups compared to baseline CORT levels in vehicle-treated rats, demonstrating its effectiveness in both sexes. In short, these data show the expected exaggerated DEX suppression of CORT in SPS-exposed males but not females. This sex difference in DEX sensitivity clearly reflects the effects of trauma exposure per se, and not resistance to DEX in females, since DEX lowered CORT levels in control females both before and after acute stress but drove only *baseline* CORT levels down in SPS-exposed females (Fig. 2b). SPS did not affect baseline CORT levels in either sex, and control females showed the expected higher baseline and post-stress CORT levels than males (Fig. 2b).

These striking sex differences in response to SPS suggested that SPS might also differentially affect cellular measures in the brain of males and females. This is indeed what we found when we examined the expression of GR and cFos in brain regions implicated in the control of the traumatic stress response [20]. While SPS moderately increased the number of GR-expressing neurons in the paraventricular nucleus of the hypothalamus (PVN) of males compared to control males (*P = 0.06*), SPS significantly *decreased* their number in females compared to control females (*P* < 0*.*0001; Fig. 2c). GR expression in the PVN of control (unstressed) females was also higher than that of control males (*P* < 0.0001; Fig. 2c). GR expression in the CA1/2 region of the dorsal hippocampus was also affected differentially by SPS in males and females, as SPS decreased the number of GR+ neurons in males compared to control males but increased their number in females compared to control females (Fig. 2d), as previously reported [14], leading to a nearly significant interaction between sex and SPS (*P* = 0.05). SPS had surprisingly little effect on the cFos response to acute restraint stress in the brains of males, including in the prelimbic (PrL) or infralimbic (IL) sugbregions of the medial prefrontal cortex (mPFC) (Fig. 2e), the basolateral amygdala (BLA) and medial amygdala (MeA; Fig. 2f, g). In contrast, SPS broadly affected the cFos response to acute stress in the brains of females, increasing the number of cFos+ neurons more than a week after exposure to trauma in both the PrL (*P* = 0.001) and IL (*P* = 0.001) (Fig. 2e), and in the *right* BLA (*P* = 0.021; Fig. 2f) compared to control females. The number of cFos+ neurons induced by acute stress was also sexually differentiated in the mPFC of control rats, with control females having fewer such neurons than control males (PrL *P* = 0.011; IL *P* = 0.042; Fig. 2e), as previously reported [29]. Additionally, body weight cannot account for these sex differences in the trauma response (Table 1) since SPS had no effect on body weight in either sex. Taken together, we found sex differences in the response to SPS in every outcome measure examined. Considering all outcome measures in a correlation matrix (see Additional file 3), there were significant correlations only between PVN GR and post-stress CORT and between mPFC-IL cFos and right BLA cFos, indicating that most measures were not correlated.

**Table 1:** SPS did not affect body weight in males or females

Group		Body weight in grams (SEM)	
		Baseline	Post-test
Male	Ctrl	297.2 (3.4)	350.0* (7.1)
	SPS	297.5 (4.9)	341.9* (7.9)
Female	Ctrl	191.5 (3.0)	213.9* (5.4)
	SPS	194.5 (3.0)	214.8* (3.2)

Rats were weighed before undergoing SPS or control conditions (baseline) and on the day of the dexamethasone suppression test (post-test). SPS did not affect body weight, and all rats gained weight over the course of the experiment (*, vs baseline). As expected, all females weighed less than males. Data presented as mean ± SEM. Significance set at *P* < .05 (indicated by asterisk) for planned pairwise comparisons (Bonferroni). Refer to Additional file 1 for full statistical results

### Predator exposure (PredX) induces comparable sex differences in ASR and DST

**Fig. 3:**
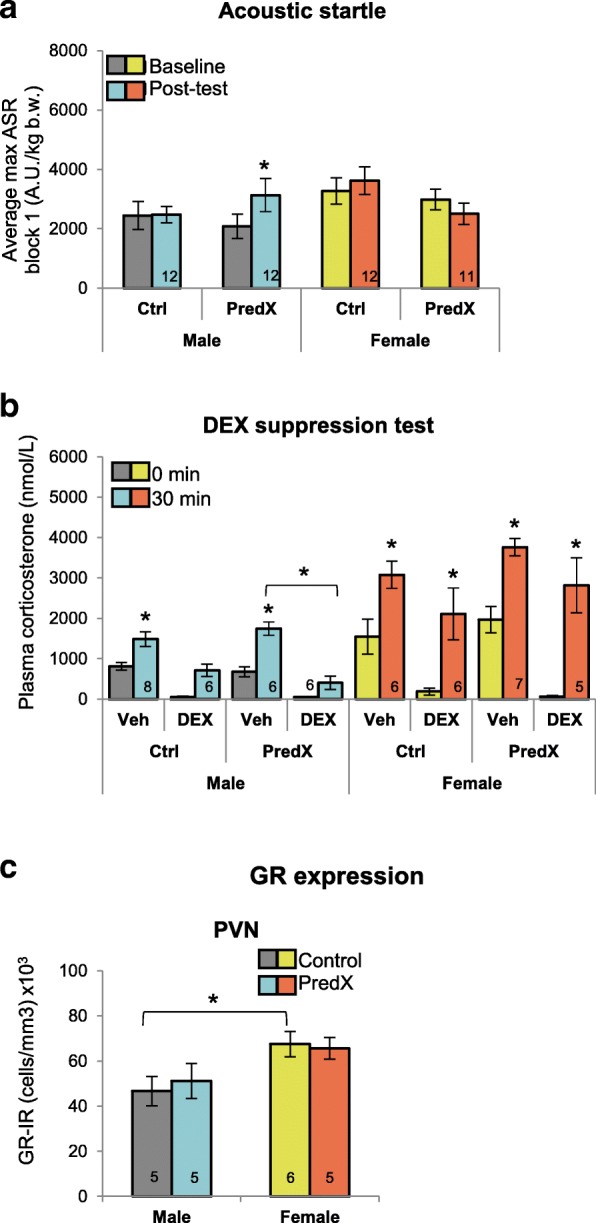
PredX leads to comparable sex differences in ASR and negative feedback control of CORT. **a** Only males and not females show an enhanced ASR after PredX exposure, replicating the sex difference found after SPS exposure (Fig. 2a). **b** Likewise, PredX enhanced HPA negative feedback in males but not females. DEX blocked the stress-induced increase in CORT levels *only* in PredX males, indicating an enhanced sensitivity to DEX in this group and not in PredX females, paralleling results in the SPS model (Fig. 2b). As expected, CORT levels were significantly higher in female compared to males. Again, DEX lowered baseline CORT levels (0 min) to near zero in all groups, demonstrating the effectiveness of DEX in both sexes. **c** Unlike SPS, PredX did not affect GR expression in the PVN of either sex, although the baseline sex difference was replicated (see Fig. 2c); females have more GR+ neurons in the PVN than males. These data suggest that GR expression in the PVN may be responsive to only some types of stress. Data are presented as mean ± SEM. Significance set at *P* < .05 (indicated by asterisk) for planned pairwise comparisons (Bonferroni). Refer to Additional file 2 for full statistical results

To test the generality of this sex difference in the trauma response, we tested adult male and female rats in a different model of PTSD, the PredX model. Rats were exposed to a live female cat for 1 h. Like SPS, PredX enhanced both ASR (*P* = 0.016 SPS male post-test compared to baseline; Fig. 3a) and DEX suppression of CORT (Fig. 3b) for males but not females. Specifically, DEX blocked an increase in CORT levels for PredX-exposed males, leading to a significant difference in CORT levels between DEX and vehicle-treated PredX males (*P* = 0.010) but not for control males. Like for SPS, this same trauma-specific effect was not seen in PredX-exposed females, again showing that prior exposure to a traumatic event enhances sensitivity to DEX in males but not in females. Females again displayed the expected higher baseline and post-restraint CORT levels compared to males (Fig. 3b), and DEX lowered baseline CORT levels in all four groups (Fig. 3b), again confirming the effectiveness of the DEX treatment. Females again had higher GR expression in the PVN than males, but unlike SPS, PredX did not affect GR expression in the PVN of either sex (Fig. 3c). Such dissociations in the stress response across different stressors help to identify core traits of the sex-specific phenotype that are independent of stressor type. The effect of trauma on GR expression in the PVN may be stressor specific. Overall, the pattern of sex differences was remarkably similar after exposure to PredX and SPS, and again, occurred independent of effects on body weight (Table 2).

**Table 2:** PredX did not affect body weight in males or females

Group		Body weight in grams (SEM)	
		Baseline	Post-test
Male	Ctrl	277.1 (3.8)	314.5* (5.2)
	PredX	273.2 (3.9)	306.2* (4.8)
Female	Ctrl	175.8 (2.3)	191.9* (2.4)
	PredX	176.1 (1.7)	192.9* (2.0)

Rats were weighed before undergoing PredX or control conditions (baseline) and on the day of the dexamethasone suppression test (post-test). PredX did not affect body weight and all rats gained weight over the course of the experiment (*, vs baseline). As expected, all females weighed less than males. Data presented as mean ± SEM. Significance set at *P* < .05 (indicated by asterisk) for planned pairwise comparisons (Bonferroni). Refer to Additional file 2 for full statistical results

Taken together, these data establish sex-specific responses to traumatic stress that are independent of the type of stressor, showing that the widely presumed core traits of PTSD may only apply to males. Such data also indicate that there are fundamental sex differences in the neurobiology underlying the traumatic stress response.

## Discussion

Sex differences in the traumatic stress response are among the most widely reported phenomena in epidemiological and clinical studies, but the neurobiological basis for these differences is unknown, largely due to an overwhelming male bias in preclinical research [22]. We find robust and novel sex differences in the traumatic stress response. Only male rats showed a hyper-responsive phenotype with an enhanced ASR and exaggerated negative feedback control of the stress hormone response, both core attributes of PTSD in men [21, 39]. SPS did not affect baseline CORT in either sex, as previously reported for males [36], further suggesting that the effect of trauma is on the negative feedback control of the HPA axis in males. These same measures suggest no effect of trauma in females, but brain measures indicated otherwise.

For example, we found that SPS exposure nearly 2 weeks earlier increased cFos activity in the mPFC and in the right BLA of females but not males. Based on lesion and imaging studies, the right amygdala is uniquely implicated in contextual fear conditioning in rats [42] and humans with PTSD [43, 44] such that the right amygdala is dominant over the left amygdala in emotional processing and fear learning. In humans with PTSD, symptom severity is positively correlated with regional cerebral blood flow to the right, but not left, amygdala [45, 46]. Whether this amygdalar lateralization is due to lateralized neurocircuitry and/or neurochemical differences is not yet known. Neural projections from the mPFC to the BLA, which are involved in conditioned fear responses, are thought to be disrupted in PTSD and are differentially recruited in male and female rats during fear conditioning and extinction [12]. Thus, our findings of female-specific activation in the mPFC and BLA during acute restraint stress that reminds females of a prior trauma experience could reflect these sex-specific mechanisms of fear conditioning and extinction.

We also observed diametrically opposed effects of trauma on GR expression in the PVN and CA1/2 region of the dorsal hippocampus of males and females (Fig. 2c, d), conforming to the opposing effects of stress in males and females in other rodent studies [47–50]. In response to SPS, we found that females exhibited increased GR expression in the CA 1/2 region of the dorsal hippocampus while males showed the opposite. These results corroborate the only other study examining sex differences in this model, which found that these sex differences in GR expression correlated with sex differences in fear extinction retention [14]. The sex-specific response of decreased GR expression in the female PVN likely alters the sensitivity of the PVN to stress hormones and may explain why trauma-exposed males show enhanced negative feedback control of the HPA axis, while trauma-exposed females do not. However, changes in GR in the PVN cannot be the only factor contributing to the divergent effects of trauma on negative feedback control of the CORT response since PredX had no effect on GR expression in the PVN of either sex but had the same divergent effects on the HPA axis. Nonetheless, PTSD patients have an increased number of GR-expressing blood lymphocytes and increased sensitivity to glucocorticoids [51, 52], suggesting that measures of GR expression in blood may well reflect the effects of traumatic stress on GR expression in the brain; however, it should be noted that these populations included predominately males and any sex-specific effects of traumatic stress on GR have not been investigated.

While it seems that the same neural correlates (e.g., hippocampus, mPFC, and amygdala) are enlisted to manage stress in males and females, the specific mechanisms involved are fundamentally different [12, 53]. The robust sex differences found in some measures of control rats (e.g., females have higher baseline and post-stress CORT levels, higher GR expression in the PVN, and lower cFos expression in the mPFC after acute restraint) have been previously reported and may well predispose males and females to respond differently to traumatic stress [29, 30, 54, 55].

A recent study indicates that trauma does not in general affect the cFos response of the mPFC and amygdala of males [56], consistent with our own results. Interestingly, however, this study identified a small minority (15%) of males that responded to trauma with *increased* cFos expression in both IL mPFC and BLA, similar to what we see in females exposed to SPS (Fig. 2e, f), and this cFos response was associated with anhedonic behavior [56]. These data suggest mPFC and amygdala activation may be relevant markers of trauma pathology for females but generally not males and may be associated with an anhedonic behavioral phenotype in females. Future studies would benefit by including behavioral measures of anhedonia that might better illuminate the nature of the sex difference in the traumatic stress response. Indeed, a depressive-like phenotype in SPS-exposed females is suggested by the decreased sensitivity to DEX and decreased GR expression in the PVN we report here, as depressed patients show decreased DEX sensitivity and decreased lymphocyte GR expression [51]. Taken together, the implication of our findings is that the trauma response in females may share common attributes of depression, characterized by increased mPFC and amygdala activity, blunted ASR [57], and *reduced* rather than increased HPA negative feedback [39, 58]. Indeed, the traumatic stress response in humans is sex-biased, as men with PTSD show more externalizing symptoms (e.g., hyperarousal, aggression, and risk-taking behaviors), while women with PTSD show more internalizing symptoms (e.g., sadness, loss of pleasure, and social difficulties) [7, 9, 59, 60]. We also recently discovered sex-specific effects of SPS on sucrose preference and social interaction, common measures of depressive-like behavior, *only* in female rats and not in males (see companion paper), further implicating a depressive-like phenotype of the female trauma response. These results suggest an important consideration: if only outcome measures developed in males are used to detect the effects of traumatic stress in females, erroneous conclusions will likely be drawn.

Indeed, based only on the ASR and DST, females might appear more resilient than males to the deleterious effects of traumatic stress, but as we looked further, it became clear that females are not necessarily more resilient but rather, respond *differently* to trauma. This is reminiscent of other effects of stress on males and females. For example, in response to uncontrollable footshocks, females appear more resilient than males to the negative effects of stress on learning that involves operant conditioning [61], but if classical conditioning is involved, females are less resilient than males [62], suggesting that sex may not be a factor that confers susceptibility or resilience to stress per se but simply leads to different responses. Clearly, the choice of outcome measures can determine the conclusions drawn. Our data suggest that the line between resiliency and susceptibility is not as clear as presumed [63], largely because whether an individual is judged as susceptible is tied to the measures used (Additional file 4). Moreover, an individual may be resilient in one domain of functioning but not in another, or at one time during the lifespan but not another [64]. Put more simply, female rats in these studies can be said to be resilient to trauma only if one starts with the presumption that only male-typical responses to trauma are valid.

While our data seem to question the heuristic value of the terms “resilience” and “susceptibility,” these terms remain useful and valid. Not only does the epidemiological data tell us that only a small fraction of people exposed to trauma develop PTSD, indicating that differences in susceptibility are real, but also a growing list of identified gene polymorphisms and epigenetic states appear to bias the nervous system toward more or less resilience against the negative effects of stress. Nonetheless, our data suggest that the use of these terms may be defined differently when sex is considered.

## Conclusions

In sum, we are the first to describe distinct responses to traumatic stress in two different animal models linked to a single biological factor: sex. We propose that such sex differences reflect differences in the underlying neurobiology. This conclusion has wide reaching implications for therapies that will effectively treat PTSD in *both* men and women. Factors that mediate differences in how individuals adjust after traumatic stress are attractive targets for the prevention and treatment of PTSD. At least 75% of people in the USA experience a traumatic event in their lifetime and do *not* develop PTSD [65], begging the question of *why some people who experience trauma develop PTSD while others do not?* The significant sex differences in the prevalence of stress-related disorders in humans is a call for inquiries into the factors behind such differences, which will undoubtedly offer insight into the brain regions and mechanisms that underlie individual differences in the response to traumatic stress.

## Additional files

Additional file 1: Statistical results for data shown in Fig. 2 and Table 1. All pairwise comparisons use Bonferroni adjustment for multiple comparisons. RM denotes repeated measure; otherwise assume between group measures. Only statistically significant pairwise comparisons are shown. (DOCX 25 kb)

Additional file 2: Statistical results for data shown in Fig. 3 and Table 2. All pairwise comparisons use Bonferroni adjustment for multiple comparisons. RM denotes repeated measure; otherwise assume between group measures. Only statistically significant pairwise comparisons are shown. (DOCX 21 kb)

Additional file 3: Descriptive statistics and correlation matrix for SPS study variables. **Correlation is significant at the 0.01 level (2-tailed); *Correlation is significant at the 0.05 level (2-tailed). (DOCX 17 kb)

Additional file 4: Susceptibility and resilience are a function of the measure considered. Even within males, different measures of traumatic stress indicate different degrees of vulnerabilities to traumatic stress within a given individual. Note that for one SPS-exposed male (red triangle), the acoustic startle response (ASR) is markedly elevated indicating a severe effect of SPS on this male. However, based on the dexamethasone (DEX) suppression test (DST), this same male is minimally affected by SPS. Moreover, glucocorticoid receptor (GR) expression in the paraventricular nucleus of the hypothalamus (PVN) indicates that SPS had a moderate effect on this same male. Other individual males exposed to SPS also show marked variability across these three measures, underscoring the idea that like many psychological phenomena, accurate diagnosis of PTSD requires a composite view of numerous diagnostic measures. Values for all individuals are indicated by the colored circles, with the mean (black larger circle) and standard deviation (black error bars) of these values. (DOCX 112 kb)

## Abbreviations

ASR: Acoustic startle response
BLA: Basolateral amygdala
CORT: Corticosterone
DEX: Dexamethasone
DST: Dexamethasone suppression test
GR: Glucocorticoid receptor
HPA: Hypothalamic-pituitary-adrenal
IHC: Immunohistochemistry
IL: Infralimbic region of the medial prefrontal cortex
MeA: Medial amygdala
mPFC: Medial prefrontal cortex
PredX: Predator exposure
PrL: Prelimbic region of the medial prefrontal cortex
PTSD: Post-traumatic stress disorder
PVN: Paraventricular nucleus of the hypothalamus
SPS: Single prolonged stress
