# Supplementary material for: Sex differences in the traumatic stress response: PTSD symptoms in women recapitulated in female rats
Source: Biol Sex Differ. 2018 Jul 5;9:31. doi: 10.1186/s13293-018-0191-9 (PMC6034295; doi:10.1186/s13293-018-0191-9)
Supplement: Supplementary file 1 — Statistical results for data shown in Fig. 2 and Table 1. All pairwise comparisons use Bonferroni adjustment for multiple comparisons. RM denotes repeated measure; otherwise assume between group measures. Only statistically significant pairwise comparisons are shown. (DOCX 25 kb) [file 13293_2018_191_MOESM1_ESM.docx]

| **Additional file 1. Statistical results for data shown in Figure 2 and Table 1. All pairwise comparisons use Bonferroni adjustment for multiple comparisons. RM denotes repeated measure; otherwise assume between group measures. Only statistically significant pairwise comparisons are shown.** | | | | |  |
| --- | --- | --- | --- | --- | --- |
| *Outcome measure* | *Statistical test* | *Main effects/interactions* | *p value* | *Power (α=0.05)* | *Partial eta^2^* |
| **ASR**  **(Fig. 2a)** | RM 3-way ANOVA (stress*sex*time) | Main effect: time (*F*­_1,44_=2.286) | 0.049 | 0.316 | 0.049 |
|  |  | Main effect: stress (*F*­_1,44_=0.037) | 0.848 | 0.054 | 0.001 |
|  |  | Main effect: sex (*F*­_1,44_=0.487) | 0.489 | 0.105 | 0.011 |
|  |  | Interaction: time*sex (*F*­_1,44_<0.0001) | 0.989 | 0.050 | 0.000 |
|  |  | Interaction: stress*time (*F*­_1,44_=4.324) | 0.043 | 0.529 | 0.089 |
|  |  | Interaction: stress*sex (*F*­_1,44_=0.135) | 0.715 | 0.065 | 0.003 |
|  |  | Interaction: time*stress*sex (*F*­_1,44_=1.963) | 0.168 | 0.278 | 0.043 |
|  |  | Pairwise: SPS male time 1 v. 2 | 0.017 | 0.683 |  |
| **DST**  **(Fig. 2b)** | RM 4-way ANOVA (stress*sex*time*DEX) | Main effect: time (*F*­_1,43_=221.260) | <0.0001 | 1.000 | 0.837 |
|  |  | Main effect: DEX (*F*­_1,43_=28.931) | <0.0001 | 1.000 | 0.402 |
|  |  | Main effect: sex (*F*­_1,43_=81.192) | <0.0001 | 1.000 | 0.654 |
|  |  | Main effect: stress (*F*­_1,43_=1.302) | 0.260 | 0.200 | 0.029 |
|  |  | Interaction: time*stress (*F*­_1,43_=1.196) | 0.280 | 0.188 | 0.027 |
|  |  | Interaction: time*DEX (*F*­_1,43_=1.349) | 0.252 | .0206 | 0.030 |
|  |  | Interaction: sex*time (*F*­_1,43_=62.139) | <0.0001 | 1.000 | 0.591 |
|  |  | Interaction: time*DEX*stress (*F*­_1,43_=0.950) | 0.335 | .0159 | 0.022 |
|  |  | Interaction: time*stress*sex (*F*­_1,43_=0.078) | 0.781 | 0.059 | 0.002 |
|  |  | Interaction: sex*time*DEX (*F*­_1,43_=9.021) | 0.004 | 0.835 | 0.173 |
|  |  | Interaction: sex*time*DEX*stress (*F*­_1,43_=4.634) | 0.037 | 0.557 | 0.097 |
|  |  | Interaction: stress*DEX (*F*­_1,43_=0.859) | 0.359 | .0148 | 0.020 |
|  |  | Interaction: stress*sex (*F*­_1,43_=0.315) | 0.578 | 0.085 | 0.007 |
|  |  | Interaction: DEX*sex (*F*­_1,43_=0.447) | 0.507 | 0.100 | 0.010 |
|  |  | Interaction: stress*DEX*sex (*F*­_1,43_=1.568) | 0.217 | 0.232 | 0.035 |
|  |  | Pairwise: SPS male time 2 DEX v. veh | 0.022 | 0.639 |  |
|  |  | Pairwise: SPS female time 1 DEX v. veh | <0.0001 | 0.999 |  |
|  |  | Pairwise: control male time 1 DEX v. veh | 0.05 | 0.506 |  |
|  |  | Pairwise: control female time 1 DEX v. veh | <0.0001 | 0.995 |  |
|  |  | Pairwise: control female time 2 DEX v. veh | 0.019 | 0.663 |  |
|  |  | Pairwise: SPS DEX time 2 male v. female | <0.0001 | 1.000 |  |
|  |  | Pairwise: SPS vehicle time 1 male v. female | 0.001 | 0.953 |  |
|  |  | Pairwise: SPS vehicle time 2 male v. female | <0.0001 | 0.974 |  |
|  |  | Pairwise: control DEX time 2 male v. female | <0.0001 | 0.977 |  |
|  |  | Pairwise: control vehicle time 1 male v. female | 0.006 | 0.802 |  |
|  |  | Pairwise: control vehicle time 2 male v. female | <0.0001 | 1.000 |  |
|  |  | Pairwise: SPS DEX female time 1 v. 2 | <0.0001 | 1.000 |  |
|  |  | Pairwise: SPS vehicle male time 1 v. 2 | 0.002 | 0.886 |  |
|  |  | Pairwise: SPS vehicle female time 1 v. 2 | <0.0001 | 1.000 |  |
|  |  | Pairwise: control DEX male time 1 v. 2 | 0.019 | 0.665 |  |
|  |  | Pairwise: control DEX female time 1 v. 2 | <0.0001 | 1.000 |  |
|  |  | Pairwise: control vehicle male time 1 v. 2 | 0.001 | 0.943 |  |
|  |  | Pairwise: control vehicle female time 1 v. 2 | <0.0001 | 1.000 |  |
| **PVN GR (Fig. 2c)** | 2-way ANOVA (stress*sex) | Main effect: stress (*F*­_1,18_=4.895) | 0.040 | 0.553 | 0.214 |
|  |  | Main effect: sex (*F*­_1,18_=43.023) | <0.0001 | 1.000 | 0.705 |
|  |  | Interaction: stress*sex (*F*­_1,18_=26.834) | <0.0001 | 0.998 | 0.599 |
|  |  | Pairwise: control male v. female | <0.0001 | 1.000 |  |
|  |  | Pairwise: female SPS v. control | <0.0001 | 0.999 |  |
|  |  | Pairwise: male SPS v. control | 0.060 | 0.477 |  |
| **CA1/2 GR**  **(Fig. 2d)** | 2-way ANOVA  (stress*sex) | Main effect: sex (*F*­_1,16_=0.002) | 0.963 | 0.050 | 0.000 |
|  |  | Main effect: stress (*F*­_1,16_=0.002) | 0.970 | 0.050 | 0.000 |
|  |  | Interaction: sex*stress (*F*­_1,16_=4.682) | 0.050 | 0.517 | 0.265 |
| **PrL cFos (Fig. 2e)** | 2-way ANOVA (stress*sex) | Main effect: sex (*F*­_1,24_=0.122) | 0.730 | 0.063 | 0.006 |
|  |  | Main effect: stress (*F*­_1,24_=3.633) | 0.070 | 0.444 | 0.147 |
|  |  | Interaction: stress*sex (*F*­_1,24_=12.288) | 0.002 | 0.916 | 0.369 |
|  |  | Pairwise: SPS male v. female | 0.040 | 0.552 |  |
|  |  | Pairwise: control male v. female | 0.011 | 0.754 |  |
|  |  | Pairwise: female SPS v. control | 0.001 | 0.960 |  |
| **IL cFos (Fig. 2f)** | 2-way ANOVA (stress*sex) | Main effect: stress (*F*­_1,24_=5.422) | 0.030 | 0.603 | 0.205 |
|  |  | Main effect: sex (*F*­_1,24_=0.121) | 0.732 | 0.063 | 0.006 |
|  |  | Interaction: stress*sex (*F*­_1,24_=11.228) | 0.003 | 0.891 | 0.348 |
|  |  | Pairwise: SPS male v. female | 0.018 | 0.688 |  |
|  |  | Pairwise: control male v. female | 0.042 | 0.542 |  |
|  |  | Pairwise: female SPS v. control | 0.001 | 0.974 |  |
| **BLA cFos (Fig. 2f)** | 3-way ANOVA (stress*sex*side) | Main effect: stress (*F*­_1,38_=6.454) | 0.015 | 0.697 | 0.145 |
|  |  | Main effect: sex (*F*­_1,38_=0.001) | 0.973 | 0.050 | 0.000 |
|  |  | Main effect: side (*F*­_1,38_=1.178) | 0.285 | 0.185 | 0.030 |
|  |  | Interaction: sex*stress (*F*­_1,38_=0.304) | 0.585 | 0.084 | 0.008 |
|  |  | Interaction: sex*side (*F*­_1,38_=3.072) | 0.088 | 0.401 | 0.075 |
|  |  | Interaction: stress*side (*F*­_1,38_=0.702) | 0.407 | 0.129 | 0.018 |
|  |  | Interaction: sex*stress*side (*F*­_1,38_=0.590) | 0.447 | 0.116 | 0.015 |
|  |  | Pairwise: female SPS left v. right | 0.029 | 0.602 |  |
|  |  | Pairwise: female right SPS v. control | 0.021 | 0.650 |  |
| **MeA cFos (Fig. 2g)** | 2-way ANOVA (stress*sex) | Main effect: sex (*F*­_1,19_=2.496) | 0.131 | 0.323 | 0.116 |
|  |  | Main effect: stress (*F*­_1,19_=0.134) | 0.718 | 0.064 | 0.007 |
|  |  | Interaction: sex*group (*F*­_1,19_=0.285) | 0.600 | 0.080 | 0.015 |
| **Body wt.**  **(Table 1)** | RM 3-way ANOVA (stress*sex*time) | Main effect: sex (*F*­_1,44_=626.044) | <0.0001 | 1.000 | 0.934 |
|  |  | Main effect: time (*F*­_1,44_=467.470) | <0.0001 | 1.000 | 0.914 |
|  |  | Main effect: stress (*F*­_1,44_=0.098) | 0.756 | 0.061 | 0.002 |
|  |  | Interaction: time*stress (*F*­_1,44_=1.640) | 0.207 | 0.240 | 0.036 |
|  |  | Interaction: time*sex (*F*­_1,44_=63.62) | <0.0001 | 1.000 | 0.591 |
|  |  | Interaction: stress* sex (*F*­_1,44_=0.256) | 0.615 | 0.079 | 0.006 |
|  |  | Interaction: time*stress* sex (*F*­_1,44_=1.640) | 0.207 | 0.240 | 0.036 |
|  |  | Pairwise: SPS time 1 male v. female | <0.0001 | 1.000 |  |
|  |  | Pairwise: SPS time 2 male v. female | <0.0001 | 1.000 |  |
|  |  | Pairwise: Control time 1 male v. female | <0.0001 | 1.000 |  |
|  |  | Pairwise: Control time 2 male v. female | <0.0001 | 1.000 |  |
|  |  | Pairwise: SPS male time 1 v. 2 | <0.0001 | 1.000 |  |
|  |  | Pairwise: Control male time 1 v. 2 | <0.0001 | 1.000 |  |
|  |  | Pairwise: SPS female time 1 v. 2 | <0.0001 | 1.000 |  |
|  |  | Pairwise: Control female time 1 v. 2 | <0.0001 | 1.000 |  |
