# Supplementary material for: Sex differences in the traumatic stress response: PTSD symptoms in women recapitulated in female rats
Source: Biol Sex Differ. 2018 Jul 5;9:31. doi: 10.1186/s13293-018-0191-9 (PMC6034295; doi:10.1186/s13293-018-0191-9)
Supplement: Supplementary file 2 — Statistical results for data shown in Fig. 3 and Table 2. All pairwise comparisons use Bonferroni adjustment for multiple comparisons. RM denotes repeated measure; otherwise assume between group measures. Only statistically significant pairwise comparisons are shown. (DOCX 21 kb) [file 13293_2018_191_MOESM2_ESM.docx]

| **Additional file 3. Statistical results for data shown in Figure 3 and Table 2. All pairwise comparisons use Bonferroni adjustment for multiple comparisons. RM denotes repeated measure; otherwise assume between group measures. Only statistically significant pairwise comparisons are shown.** | | | | |  |
| --- | --- | --- | --- | --- | --- |
| *Outcome measure* | *Statistical test* | *Significant effects* | *p value* | *Power (α=0.05)* | *Partial eta^2^* |
| **ASR (Fig. 3a)** | RM 3-way ANOVA (stress*sex*time) | Main effect: time (*F*­_1,43_=1.290) | 0.262 | 0.199 | 0.029 |
|  |  | Main effect: stress (*F*­_1,43_=0.565) | 0.456 | 0.114 | 0.013 |
|  |  | Main effect: sex (*F*­_1,43_=2.287) | 0.138 | 0.315 | 0.050 |
|  |  | Interaction: time* stress (*F*­_1,43_=0.048) | 0.828 | 0.055 | 0.001 |
|  |  | Interaction: time*sex (*F*­_1,43_=2.005) | 0.164 | 0.283 | 0.045 |
|  |  | Interaction: stress*sex (*F*­_1,43_=1.318) | 0.257 | 0.202 | 0.030 |
|  |  | Interaction: stress*sex*time (*F*­_1,43_=4.775) | 0.034 | 0.570 | 0.100 |
|  |  | Pairwise: SPS male time 1 v. 2 | 0.016 | 0.688 |  |
| **DST (Fig. 3b)** | RM 4-way ANOVA (stress*sex*time*DEX) | Main effect: time (*F*­_1,42_=106.648) | <0.0001 | 1.000 | 0.717 |
|  |  | Main effect: sex (*F*­_1,42_=59.972) | <0.0001 | 1.000 | 0.588 |
|  |  | Main effect: DEX (*F*­_1,42_=49.196) | <0.0001 | 1.000 | 0.539 |
|  |  | Main effect: stress (*F*­_1,42_=1.444) | 0.236 | 0.217 | 0.033 |
|  |  | Interaction: time*stress (*F*­_1,42_=1.310) | 0.259 | 0.201 | 0.030 |
|  |  | Interaction: time*DEX (*F*­_1,42_=0.367) | 0.548 | 0.091 | 0.009 |
|  |  | Interaction: time*sex*stress (*F*­_1,42_=0.933) | 0.340 | 0.157 | 0.022 |
|  |  | Interaction: time*sex*DEX (*F*­_1,42_=3.997) | 0.052 | 0.497 | 0.087 |
|  |  | Interaction: time*stress*DEX (*F*­_1,42_=0.015) | 0.903 | 0.052 | 0.000 |
|  |  | Interaction: time*sex*stress*DEX (*F*­_1,42_=1.487) | 0.229 | 0.222 | 0.034 |
|  |  | Interaction: sex*stress (*F*­_1,42_=2.292) | 0.138 | 0.316 | 0.052 |
|  |  | Interaction: sex*DEX (*F*­_1,42_=1.849) | 0.181 | 0.265 | 0.042 |
|  |  | Interaction: stress*DEX (*F*­_1,42_=0.606) | 0.441 | 0.118 | 0.014 |
|  |  | Interaction: sex*stress*DEX (*F*­_1,42_=0.005) | 0.946 | 0.051 | 0.000 |
|  |  | Interaction: sex*time (*F*­_1,42_=25.235) | <0.0001 | 0.998 | 0.375 |
|  |  | Pairwise: PredX DEX time 2 male v. female | <0.0001 | 0.995 |  |
|  |  | Pairwise: PredX vehicle time 1 male v. female | <0.0001 | 0.992 |  |
|  |  | Pairwise: PredX vehicle time 2 male v. female | <0.0001 | 0.984 |  |
|  |  | Pairwise: control DEX time 2 male v. female | 0.007 | 0.784 |  |
|  |  | Pairwise: control vehicle time 1 male v. female | 0.012 | 0.730 |  |
|  |  | Pairwise: control vehicle time 2 male v. female | 0.001 | 0.918 |  |
|  |  | Pairwise: Male PredX time 1 DEX v. veh | 0.042 | 0.537 |  |
|  |  | Pairwise: Male PredX time 2 DEX v. veh | 0.010 | 0.752 |  |
|  |  | Pairwise: Male control time 1 DEX v. veh | 0.010 | 0.755 |  |
|  |  | Pairwise: female PredX time 1 DEX v. veh | <0.0001 | 1.000 |  |
|  |  | Pairwise: female control time 1 DEX v. veh | <0.0001 | 0.994 |  |
|  |  | Pairwise: Male PredX veh time 1 v. 2 | 0.006 | 0.801 |  |
|  |  | Pairwise: Male control vehicle time 1 v. 2 | 0.043 | 0.532 |  |
|  |  | Pairwise: female PredX DEX time 1 v. 2 | <0.0001 | 1.000 |  |
|  |  | Pairwise: female PredX vehicle time 1 v. 2 | <0.0001 | 0.999 |  |
|  |  | Pairwise: female control DEX time 1 v. 2 | <0.0001 | 0.999 |  |
|  |  | Pairwise: female control vehicle time 1 v. 2 | <0.0001 | 0.979 |  |
| **PVN GR (Fig. 3c)** | 2-way ANOVA (stress*sex) | Main effect: sex (*F*­_1,17_=7.921) | 0.012 | 0.756 | 0.318 |
|  |  | Main effect: stress (*F*­_1,17_=0.044) | 0.836 | 0.055 | 0.003 |
|  |  | Interaction: sex*stress (*F*­_1,17_=0.263) | 0.614 | 0.077 | 0.015 |
|  |  | Pairwise: Control male v. female | 0.028 | 0.621 |  |
| **Body wt.**  **(Table2)** | RM 3-way ANOVA (stress*sex*time) | Main effect: sex (*F*­_1,60_=1026.650) | <0.0001 | 1.000 | 0.945 |
|  |  | Main effect: time (*F*­_1,60_=909.046) | <0.0001 | 1.000 | 0.938 |
|  |  | Main effect: stress (*F*­_1,60_=0.666) | 0.418 | 0.127 | 0.011 |
|  |  | Interaction: time*stress (*F*­_1,60_=1.158) | 0.286 | 0.185 | 0.019 |
|  |  | Interaction: time*stress*sex (*F*­_1,60_=2.183) | 0.145 | 0.307 | 0.035 |
|  |  | Interaction: stress*sex (*F*­_1,60_=0.983) | 0.325 | 0.164 | 0.016 |
|  |  | Interaction: time*sex (*F*­_1,60_=119.370) | <0.0001 | 1.000 | 0.665 |
|  |  | Pairwise: PredX time 1 male v. female | <0.0001 | 1.000 |  |
|  |  | Pairwise: PredX time 2 male v. female | <0.0001 | 1.000 |  |
|  |  | Pairwise: Control time 1 male v. female | <0.0001 | 1.000 |  |
|  |  | Pairwise: Control time 2 male v. female | <0.0001 | 1.000 |  |
|  |  | Pairwise: PredX male time 1 v. 2 | <0.0001 | 1.000 |  |
|  |  | Pairwise: Control male time 1 v. 2 | <0.0001 | 1.000 |  |
|  |  | Pairwise: PredX female time 1 v. 2 | <0.0001 | 1.000 |  |
|  |  | Pairwise: Control female time 1 v. 2 | <0.0001 | 1.000 |  |
