# Supplementary material for: Sex differences in the traumatic stress response: PTSD symptoms in women recapitulated in female rats
Source: Biol Sex Differ. 2018 Jul 5;9:31. doi: 10.1186/s13293-018-0191-9 (PMC6034295; doi:10.1186/s13293-018-0191-9)
Supplement: Supplementary file 3 — Descriptive statistics and correlation matrix for SPS study variables. **Correlation is significant at the 0.01 level (2-tailed); *Correlation is significant at the 0.05 level (2-tailed). (DOCX 17 kb) [file 13293_2018_191_MOESM3_ESM.docx]

Additional file 3: Descriptive statistics and correlation matrix for SPS study variables.

|  | **CORT**  **(0 min)** | **CORT**  **(30 min)** | **GR**  **(PVN)** | **ASR**  **(baseline)** | **ASR**  **(post-test)** | | **cFos**  **(mPFC-IL)** | | **cFos**  **(mPFC-PrL)** | | **cFos**  **(right BLA)** | | **cFos**  **(left BLA)** | | **cFos**  **(MEA)** | | **GR**  **(hipp)** | |
| --- | --- | --- | --- | --- | --- | --- | --- | --- | --- | --- | --- | --- | --- | --- | --- | --- | --- | --- |
| **CORT (0 min)** | 1 |  |  |  |  |  | |  | |  | |  | |  | |  | |  |
| **CORT (30 min)** | .433^**^ | 1 |  |  |  |  | |  | |  | |  | |  | |  | |  |
| **GR (PVN)** | 0.185 | .583^**^ | 1 |  |  |  | |  | |  | |  | |  | |  | |  |
| **ASR (baseline)** | 0.016 | -0.108 | -0.113 | 1 |  |  | |  | |  | |  | |  | |  | |  |
| **ASR (post-test)** | 0.054 | -0.147 | -0.128 | .588^**^ | 1 |  | |  | |  | |  | |  | |  | |  |
| **cFos (mPFC-IL)** | 0.112 | 0.098 | -0.364 | -0.151 | 0.030 | 1 | |  | |  | |  | |  | |  | |  |
| **cFos (mPFC-PrL)** | 0.265 | 0.203 | -0.368 | -0.169 | 0.013 | .752^**^ | | 1 | |  | |  | |  | |  | |  |
| **cFos (right BLA)** | 0.019 | -0.038 | -0.135 | -0.353 | -0.105 | -0.034 | | 0.051 | | 1 | |  | |  | |  | |  |
| **cFos (left BLA)** | -0.084 | 0.366 | 0.005 | -0.324 | -0.085 | .447^*^ | | 0.229 | | 0.217 | | 1 | |  | |  | |  |
| **cFos (MEA)** | 0.106 | 0.258 | 0.189 | -0.028 | -0.145 | 0.179 | | 0.317 | | -0.369 | | 0.151 | | 1 | |  | |  |
| **GR (hipp CA1/2)** | -0.044 | 0.111 | -0.150 | 0.132 | -0.283 | 0.345 | | 0.185 | | 0.029 | | 0.236 | | 0.273 | | 1 | |  |
|  |  |  |  |  |  |  | |  | |  | |  | |  | |  | |  |
| Notes: **Correlation is significant at the 0.01 level (2-tailed); *Correlation is significant at the 0.05 level (2-tailed). | | | | | | | | | | | | | | | |  | |  |
