# Supplementary material for: Sex differences in the traumatic stress response: PTSD symptoms in women recapitulated in female rats
Source: Biol Sex Differ. 2018 Jul 5;9:31. doi: 10.1186/s13293-018-0191-9 (PMC6034295; doi:10.1186/s13293-018-0191-9)
Supplement: Supplementary file 4 — Susceptibility and resilience are a function of the measure considered. Even within males, different measures of traumatic stress indicate different degrees of vulnerabilities to traumatic stress within a given individual. Note that for one SPS-exposed male (red triangle), the acoustic startle response (ASR) is markedly elevated indicating a severe effect of SPS on this male. However, based on the dexamethasone (DEX) suppression test (DST), this same male is minimally affected by SPS. Moreover, glucocorticoid receptor (GR) expression in the paraventricular nucleus of the hypothalamus (PVN) indicates that SPS had a moderate effect on this same male. Other individual males exposed to SPS also show marked variability across these three measures, underscoring the idea that like many psychological phenomena, accurate diagnosis of PTSD requires a composite view of numerous diagnostic measures. Values for all individuals are indicated by the colored circles, with the mean (black larger circle) and standard deviation (black error bars) of these values. (DOCX 112 kb) [file 13293_2018_191_MOESM4_ESM.docx]

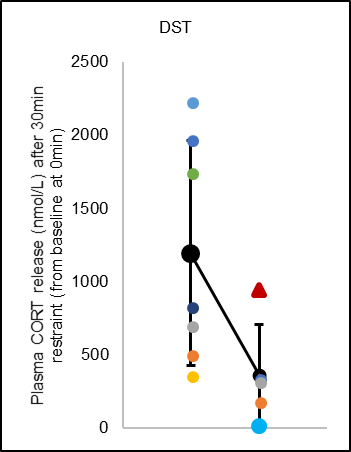

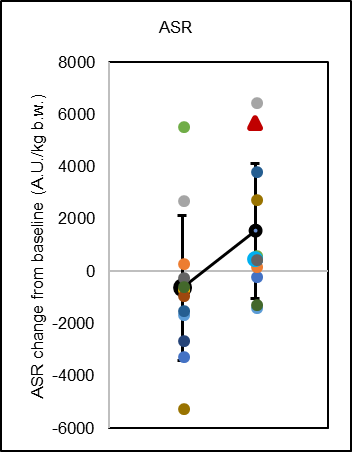

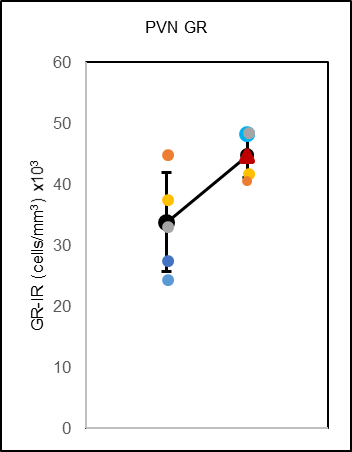


**Control**

**SPS**

**Control**

**SPS**

**Vehicle**

**DEX**

**Additional file 4. Susceptibility and resilience are a function of the measure considered.** Even within males, different measures of traumatic stress indicate different degrees of vulnerabilities to traumatic stress within a given individual. Note that for one SPS-exposed male (red triangle), the acoustic startle response (ASR) is markedly elevated indicating a severe effect of SPS on this male. However, based on the dexamethasone (DEX) suppression test (DST), this same male is minimally affected by SPS. Moreover, glucocorticoid receptor (GR) expression in the paraventricular nucleus of the hypothalamus (PVN) indicates that SPS had a moderate effect on this same male. Other individual males exposed to SPS also show marked variability across these three measures, underscoring the idea that like many psychological phenomena, accurate diagnosis of PTSD requires a composite view of numerous diagnostic measures. Values for all individuals are indicated by the colored circles, with the mean (black larger circle) and standard deviation (black error bars) of these values.
